# EKC Test of the Relationship between Nitrogen Dioxide Pollution and Economic Growth—A Spatial Econometric Analysis Based on Chinese City Data

**DOI:** 10.3390/ijerph18189697

**Published:** 2021-09-15

**Authors:** Chengyu Han, Zhaolin Gu, Hexiang Yang

**Affiliations:** 1 School of Human Settlements and Civil Engineering, Xi’an Jiaotong University, Xi’an 710049, China; pingshi3pa@163.com; 2 College of Health and Human Services, George Mason University, Fairfax, VA 22030, USA; chunrang3tan@163.com

**Keywords:** economic development, Nitrogen Dioxide pollution, spatial lag model, Environmental Kuznets Curve

## Abstract

During the just concluded 13th Five-Year Plan, China continued to maintain the momentum of rapid economic development, but still faced environmental pollution problems caused by this. Finding the relationship between Nitrogen Dioxide pollution and economic development is helpful and significant in better achieving and optimizing sustainable environmental development. Taking China’s 333 prefecture-level cities as samples from 2016 to 2018, the spatial lag model (SAR) was used to study the impact of economic growth on urban Nitrogen Dioxide pollution. The results show that Nitrogen Dioxide has strong positive characteristics of spatial spillover, but there is a linear relationship between economic growth and Nitrogen Dioxide concentration that slowly rises, and there is no inverted U-shaped relationship, which does not support the Environmental Kuznets Curve (EKC) hypothesis; The results also show the impact of per capita GDP, natural gas consumption, residential natural gas consumption, industrialization, and transportation development on the increase of Nitrogen Dioxide concentration, and the impact of green coverage on the decrease of Nitrogen Dioxide concentration. However, there is no significant relationship between technological investment and Nitrogen Dioxide concentration. The above conclusions are still valid after the robustness test, and recommendations are put forward to reduce Nitrogen Dioxide pollution.

## 1. Introduction

**Figure 1:**
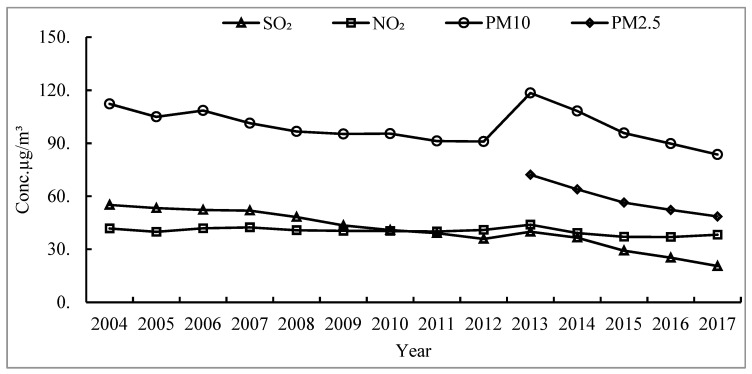
The average concentration of major air pollutants in major cities in China from 2004 to 2017.

China’s economy is developing rapidly. However, economic development is always accompanied by high-intensity energy consumption. Only three elements, natural gas, coal and oil, account for more than 85% of China’s overall energy consumption, which makes China face great environmental pressure. China implemented energy-saving and emission-reduction policies in 2013. As shown in Figure 1, we see the changes in the average concentration of major air pollutants in China’s key cities from 2004 to 2017. Since 2013, the concentrations of Sulfur Dioxide, PM_10_, and PM_2.5_ have all shown a downward trend, which proves that China has effectively alleviated some environmental air pressure after implementing energy-saving and emission-reduction policies. However, the concentration of urban Nitrogen Dioxide has not decreased, and after 2015 the figure also showed an upward trend, the reason why this article selects Nitrogen Dioxide pollutants as the research index. Although China’s methods and policies of controlling air pollutants such as Sulfur Dioxide and PM_2.5_ have become increasingly mature, the concentration of Nitrogen Dioxide cannot be effectively controlled. Although policies and technologies can effectively improve and optimize Nitrogen Dioxide emissions in the process of industrial production, the use of mobile sources on urban roads and the use of natural gas by urban residents also have a great impact on increasing urban Nitrogen Dioxide concentrations. As one of the most important pollutants in urban air, Nitrogen Dioxide causes great harm to the vironment and human health. For example, Carmona-Cabezas et al. [1] pointed out that changes in Nitrogen Dioxide concentration are closely related to changes in photochemical smog and ozone in the atmosphere. Ding et al. [2] and Lu et al. [3] pointed out that long-term living in an environment with high Nitrogen Dioxide concentration also greatly affects the human lung function and respiratory system, severely irritating the upper respiratory tract, causing shock, shortness of breath, asthma and other symptoms.

The Environmental Kuznets Curve (EKC) can effectively analyze, and we can learn from it, the relationship between economic growth and pollution emissions. Pata et al. [4] studied the relationship between renewable energy consumption and economic growth based on the EKC hypothesis; Leal and Marques [5] found an EKC relationship between economic growth and environmental degradation in countries with a high degree of globalization, but the EKC hypothesis linking economic growth and environmental degradation in countries with a low degree of globalization has not been confirmed. Hao and Liu [6] verified the EKC hypothesis between PM_2.5_ pollution and economic development in Chinese cities. However, some researchers believe that when analyzing the impact of economic growth on environmental pressures, the spatial effects of pollutants are ignored, leading to deviations in estimates. Dinda [7] stated that if pollution is not considered when analyzing air pollution emissions, the spatial effect of objects will reduce the accuracy of the analysis results. When analyzing the relationship between China’s PM_2.5_ and economic growth, Xie et al. [8] added a spatial autoregressive model to more accurately analyze the problem of haze pollution on a temporal and spatial scale. Based on the expansion of the stochastic impact study on population, wealth and technology (stirpat) framework, this paper uses data from 2016 to 2018 in 333 cities to investigate the nonlinear relationship between economic growth and Nitrogen Dioxide concentration. The spatial measurement model was compared to test the effect of urban Nitrogen Dioxide concentration, and it was found that there is no EKC relationship between Nitrogen Dioxide concentration and economic development in China. As far as we know, predecessors have mostly studied the impact of China’s economic growth on carbon dioxide, PM_2.5_, PM_10_, and Sulfur Dioxide pollution. As China has matured in the treatment of these forms of air pollution, Nitrogen Dioxide has to a certain extent become the primary air pollution problem that China needs to consider at present. This research is the first to explain the long-term effects of the correlation coefficients that affect Nitrogen Dioxide pollution based on the Quasi-maximum likelihood (QML) dynamic test model, in order to make a more accurate and comprehensive review and analysis of this research field, and to strive to fill in the gaps.

## 2. Literature Review

Wu et al. [9] have discussed the relationship between economic development and environmental pollution. Most of the research is based on the EKC hypothesis proposed by Grossman and Krueger [10]. This hypothesis believes that the impact of economic growth on pollutants is in an inverted U-shape. In the early stages of development, economic growth will lead to an increase in pollutants and the environment will continue to deteriorate. When the level of economic development reaches the turning point of EKC, as the economy continues to increase, pollutants are reduced and environmental quality is improved. There are many studies dedicated to researching and testing the EKC hypothesis, such as Gill et al. [11] and Kharbach and Chfadi [12], but there are different opinions on the existence of EKC, different research areas and different directions. The results all display certain differences, and no consensus has yet been reached yet.

Some researchers support the inverted U-shaped relationship between economic growth and environmental pollution, which has been explained by the EKC model. For example, Zhao et al. [13] analyzed the relationship between urbanization and the ecological environment in the Yangtze River Delta, observed obvious EKC effects, and established an improved EKC model that couples urbanization and the ecological environment. Thompson [14] analyzed the impact of water abundance on EKC, and the results showed that water abundance has a great impact on the turning point of EKC. When Danish et al. [15] studied the relationship between nuclear energy and carbon dioxide emissions, they verified that nuclear energy reduces environmental pollution, which highlights that more nuclear power generation in India’s energy system will help mitigate climate change. The results further show that the overall impact of population density on the IPAT equation stimulates carbon emissions. Both nuclear energy and population density contribute to the formation of the EKC curve. Adeel-Farooq et al. [16] analyzed the relationship between methane emissions and economic growth in six countries of the Association of Southeast Asian Nations from 1985 to 2012. The survey results show that the EKC assumption of CH4 emissions in these economies has proven to be valid.

Unlike the above conclusions, some studies are not consistent with the EKC hypothesis. Park studied and examined the Environmental Kuznets Curve (EKC) hypothesis and analyzed annual panel data of 16 metropolitan areas in South Korea over the past 16 years. The analysis results illustrate that the EKC relationship between Sulfur Dioxide and Nitrogen Dioxide does not have a uniform shape. Each area has its own EKC, such as U-shaped curves and inverted N-shaped curves. They believe that environmental policies should take into account the different characteristics of different regions and different types of pollutants. Bimonte and Stabile [17] proposed a reverse EKC case analysis of Italian land consumption and income. Sinha et al. [18] discussed the relationship between energy use isolation and environmental degradation in N-11 countries, and showed that for these countries the relationship between income and carbon emissions is an N-shaped model, not an inverted U-shaped model. After studying the relationship between Australian GDP and carbon dioxide, Özden and Beşe [19] did not find a significant relationship between GDP and the square of GDP and carbon dioxide, indicating that the EKC hypothesis is not valid.

In order to test the EKC hypothesis, many pollutants such as carbon dioxide, greenhouse gases, Sulfur Dioxide and industrial wastes have been widely discussed. However, there are few studies on the relationship between Nitrogen Dioxide concentration and economic growth. As mentioned in the previous article, since the concentration of Nitrogen Dioxide in China has not declined in recent years, it has been difficult to control. This aspect is worth investigating. This article attempts to fill this gap and further accurately and comprehensively evaluate the relationship between these two aspects. Empirically, due to the lack of energy statistics on this scale, little attention has been paid to city-level analysis. This data set covers 333 major cities from 2016 to 2018. It is the largest data set used in a study on Nitrogen Dioxide concentration and economic growth and provides the latest, most sufficient and more complete evidence to prove the potential variables in examining the relationship between current economic growth and the concentration of Nitrogen Dioxide. In order to better reduce the deviation of the omitted variables, industrialization, technology investment, green coverage, transportation development, natural gas consumption, and residential natural gas consumption are also used as control variables.

## 3. Research Methods

The Kuznets curve is a theory used by economist Kuznets [20] to analyze the relationship between per capita income level and distribution equity in the 1950s. The Environmental Kuznets Curve is a cubic function fitting model with economic indicators as the horizontal axis and environmental indicators as the vertical axis. The expression is:

(1) $$Y=b_{0}+b_{1}A+b_{2}A^{2}+b_{3}A^{3}$$

Among the represent letters, *Y* is an environmental indicator, and the logarithm of the concentration of Nitrogen Dioxide is selected in this article. *A* is an economic indicator, and this paper selects the logarithm of per capita GDP for calculation. *b*_0_ is a constant term, and *b*_1_, *b*_2_, and *b*_3_ are the coefficients.

Because the traditional EKC model can only reflect the relationship between economic development and environmental pollution when analyzing the relationship between the regional economy and the environment, which has a lot to do with its single structure, the EKC model does not consider the influence of other explanatory variables. When studying the relationship between the economy and the environment in a larger area, the influence of various indicators must be fully considered before a comprehensive conclusion can be drawn regarding the study area. According to the research of Özden and Beşe [21], the traditional STIRPAT model was adopted as the basic theory and analysis framework. This model not only includes environmental and economic indicators, but can also be extended to add more indicator types. This is useful for studying economic growth and the EKC relationship of environmental pollution has greatly supplemented and helped in this. The model expression is:

(2) $$I=aP^{b}A^{c}T^{d}e$$

*I* represents environmental pressure, and *P*, *A* and *T* represent population, wealth and technological level. *a*, *b*, *c*, *d* are the corresponding parameters. e represents the random error term.

In this study, the indicator of Nitrogen Dioxide concentration in each city represents environmental pressure, and per capita GDP represents the indicator of affluence. As mentioned in the literature review, previous research often incorporates the quadratic term of income and its power term into Equation (2), thus smoothing the traditional STIRPAT model into a logarithmic form, and the new model can be written as

(3) $$lnNO_{2}=a_{1}lnPCG+a_{2}lnPCG^{2}+BlnZ^{\prime }+e$$

*lnNO*_2_ is the logarithm of the concentration of Nitrogen Dioxide. *lnPCG* and *lnPCG*^2^ represent the logarithm of GDP per capita and its square term. *Z*′ represents a collective expression of other relevant variables. *a*_1_, *a*_2_ and *B* represent the coefficients of each variable. *e* represents the random error term.

**Figure 2:**
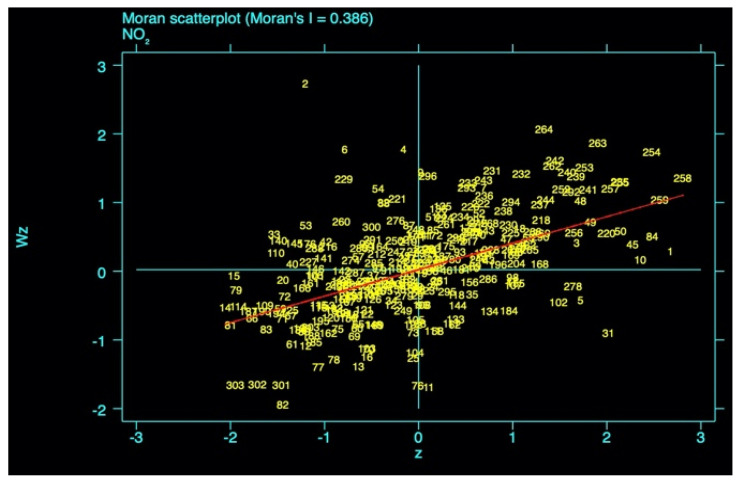
Moran index and scatter plot of Nitrogen Dioxide concentration in 333 cities of China in 2016. *p* = 0.0000.

Because Nitrogen Dioxide has significant spatial characteristics such as diffusion, accumulation, and migration in various regions, the spatial effect of Nitrogen Dioxide concentration is first analyzed. The Moran index (Moran’s I) and its scatter plot are usually used to analyze the characteristics of spatial correlation. The Moran index greater than zero indicates a positive spatial correlation. The larger the value, the more obvious the spatial correlation; the Moran index of less than zero indicates a spatial negative correlation. The smaller the value, the greater the spatial difference; when the Moran index is zero, the space becomes random. In this study, using stata software and taking the cross-sectional data of 2016 as an example, the Moran index and its scatter plot of Nitrogen Dioxide concentration were obtained. Figure 2 is the result.

According to Moran’s I statistics, the *p* value is equal to 0.0000 which is much lower than 1%, confirming that the concentration of Nitrogen Dioxide shows a very significant autocorrelation spatial characteristic. From the results of the scatter plot in Figure 2, more than 240 cities appear in the first and third quadrants of the coordinate system, which shows that cities with a high concentration of Nitrogen Dioxide are more likely to diffuse Nitrogen Dioxide to neighboring cities, and the neighboring areas of cities with low concentrations of Nitrogen Dioxide also exhibit low concentrations. At the same time, Moran’s I is 0.386, which confirms that the distribution of Nitrogen Dioxide concentration in different urban areas shows positive spatial aggregation characteristics. The concept of spatial measurement needs to be introduced in the investigation and analysis. For example, Xie et al. [8] used the spatial measurement model to optimize the analysis method of this problem, and more accurately clarified the relationship between PM_2.5_ concentration and economic growth. This research introduces the spatial lag model, and the new formula can be written as

(4) $$lnNO_{2}=\rho W_{ij}lnNO_{2}+a_{1}lnPCG+a_{2}lnPCG^{2}+BlnZ^{\prime }+v_{t}+e$$

In Equation (4), *ρ* is the spatial autocorrelation coefficient, *W* is the spatial weight matrix, and *v_t_* is the time fixed effect.

(5) $$W_{ij}=\{\begin{array}{c} 1\;,\;IF\;i\;and\;j\;adjacent\;\\ 0\;,\;IF\;i\;and\;j\;nonadjacent \end{array}$$

The establishment of the spatial weight matrix *W* is based on the mutual geographic relationship of 333 cities, and *i* and *j* are two elements in the weight matrix, representing two cities respectively, where 1 means that the two cities are adjacent to each other, and 0 means that the two cities are not adjacent. To choose a spatial lag model, first we use the LM and LR test methods to illustrate the process of choosing which spatial autoregressive model. Based on the spatial measurement problem, the three most commonly used models are SEM, SAR, and SDM models. There are also differences in the model application of different case studies and data.

According to Table 1, Moran’s I rejected the original hypothesis, indicating that it has significant spatial autocorrelation. The results of 2 LM tests and 2 robust LM tests rejected the null hypothesis, and the test results proved the existence of spatial error effects and spatial lag effects. For the hypothesis that the spatial Durbin model can be simplified to a spatial lag model or a spatial error model, the LR test in Table 2 can help verify this.

The test results of Table 2 show that the null hypothesis is not rejected, and the spatial Dubin model can be simplified to a spatial lag model or a spatial error model. For the introduction of other variables, the empirical model of Xie et al. [8] takes population density, industrialization process, technological innovation, green coverage, geographic location of city samples, urbanization, and the number of cars as control variables. When Balado-Naves et al. [22] studied the relationship between carbon dioxide emissions and economic growth, the control variables included renewable energy, the added value of tertiary industry, energy intensity, and technological progress. When Sun et al. [23] studied the relationship between China’s economic growth and carbon emissions, they introduced the indicator of solar energy technology. Based on previous studies and taking into account the data relevance and availability at the city level, industrialization, technological investment, green coverage, transportation development, natural gas consumption, and residential natural gas consumption are the control variables of this study. Based on the above considerations, the specific expression of Model 6 is

(6) $$lnNO_{2i}=\rho W_{ij}lnNO_{2i}+a_{1}lnPCG_{i}+a_{2}lnPCG_{i}^{2}+B_{1}lnSI_{i}+B_{2}lnIT_{i}+B_{3}lnGC_{i}+B_{4}lnVN_{i}+B_{5}lnNG_{i}+B_{6}lnRNG_{i}+v_{t}+e$$

*SI* stands for the process of industrialization and is expressed by the proportion of secondary industry. *IT* stands for technology investment, calculated as the ratio of technology investment to the city’s GDP. *GC* stands for green coverage, calculated according to the proportion of urban green area to the total area. *VN* stands for transportation development and is expressed by the number of cars. *NG* stands for natural gas consumption, expressed in terms of the total annual natural gas consumption of the city. *RNG* stands for residential natural gas consumption, expressed in terms of annual natural gas consumption by urban residents. *i* represents the city. The study of Xie et al. [8] introduced the variable of population number, and at the same time, taking into account the difference in winter heating between northern and southern cities in China, the dummy variable of urban geographic location was introduced, which is 1 for northern cities and 0 for southern cities. Resident natural gas consumption in this study can effectively replace the two indicators of population and urban geographic location, because the impact of population on the concentration of Nitrogen Dioxide is mainly derived from the use of natural gas by residents, which more accurately reflects the concentration of Nitrogen Dioxide by urban population impact. In addition, this compensates for the problem of introducing dummy variables caused by the difference in the geographical location of cities in the north and south of China, such as the fact that cities with large populations in the south use less natural gas, or those in northern cities use less natural gas, and those in northern cities require the introduction of dummy variables.

## 4. Data

The Nitrogen Dioxide data for 333 cities from 2016 to 2018 were obtained from historical data from the China Environmental Monitoring Center (CNEMC) (http://www.cnemc.cn/, accessed on 29 December 2020). Because the historical data of CNEMC is recorded from the daily Nitrogen Dioxide concentration of each city, we downloaded the data and calculated the annual average Nitrogen Dioxide concentration of each city in Excel. From 2016 to 2018, data on per capita GDP, industrialization, investment in technology, green coverage, transportation development, natural gas consumption, and residential natural gas consumption are from the China City Statistical Yearbook (CCSY) (http://www.stats.gov.cn/, accessed on 29 December 2020).

**Figure 3:**
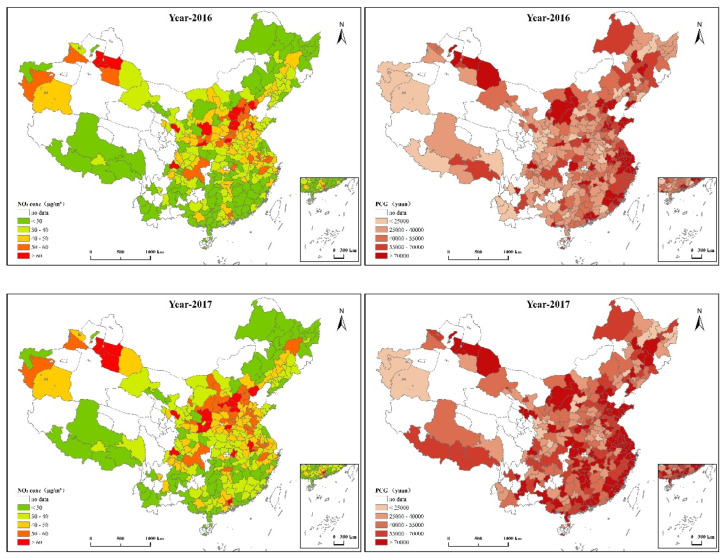

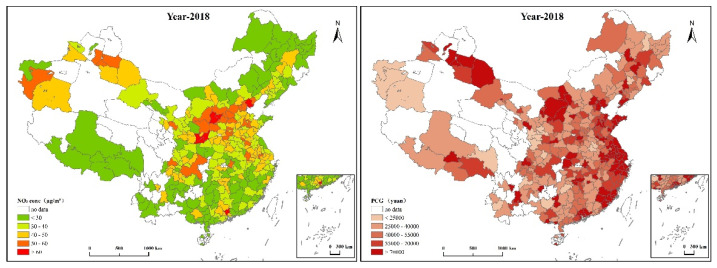
Spatial distribution of GDP level and Nitrogen Dioxide concentration of cities in China from 2016 to 2018.

Table 3 shows that China’s per capita GDP has a serious regional imbalance, with maximum and minimum values of 215,488 and 10,707 respectively. The city with the highest concentration of Nitrogen Dioxide is more than ten times higher than the city with the lowest. China’s investment in technology is very low. The average green coverage rate in Chinese cities can reach nearly 40%, and secondary industry in some heavy industrial cities accounts for more than 70%. The number of vehicles in some developed areas exceeds 5 million, which is much higher than the average. These cities are affected by more Nitrogen Dioxide pollution caused by automobile exhaust. Very few cities do not use natural gas. Figure 3 depicts the spatial distribution of Nitrogen Dioxide concentration and per capita GDP growth in different cities. The results showed that the concentration of Nitrogen Dioxide did not decrease significantly from 2016 to 2018. The concentration of Nitrogen Dioxide in central and eastern cities is higher than that in other regions, showing an obvious spatial agglomeration pattern. The three regions with high energy use in China are the Beijing-Tianjin-Hebei region, which includes Beijing, Tianjing and Hebei provinces; Fenwei plain, including Shaanxi and Shanxi; and the middle and lower reaches of the Yangtze River, including Hubei, Hunan, Jiangxi, Anhui, Jiangsu, Zhejiang and Shanghai. These three areas are more polluted by Nitrogen Dioxide. This is closely related to the large number of heavy industrial cities in this region, such as Yan’an, Cangzhou, and Tangshan. However, some areas have low PM2.5 pollution, but high income levels, such as some coastal cities such as Fuzhou and Putian, and some cities in Inner Mongolia such as Wuhai and Ordos. Therefore, it can be inferred that economic growth may have mixed and complex effects on Nitrogen Dioxide concentration.

## 5. Results and Discussion

### 5.1. Estimated Results

**Figure 4:**
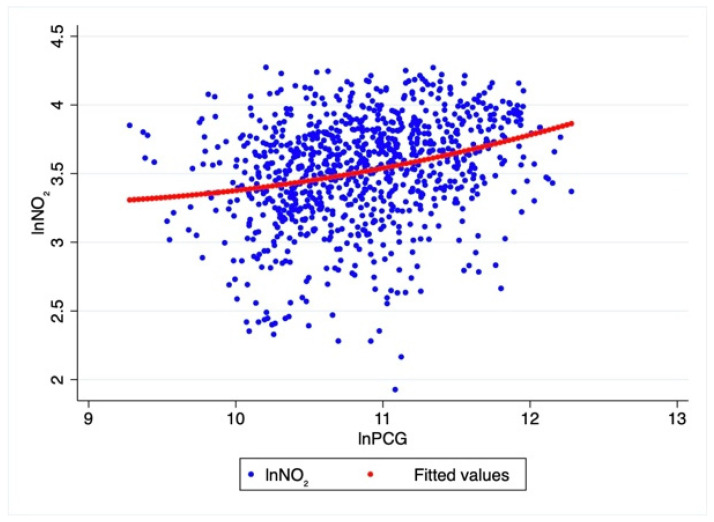
Regression curve of logarithm of Nitrogen Dioxide concentration and logarithm of PCG.

This part first estimates the logarithm of PCG and Nitrogen Dioxide concentration based on Equation (1) by EKC regression curve estimation, and explains the statistical results of the spatial lag Equation (4). Figure 4 is the regression curve of the logarithm of PCG and the logarithm of Nitrogen Dioxide concentration.

Figure 4 shows that the logarithm of Nitrogen Dioxide concentration and the logarithm of PCG do not conform to the KEC relationship, and do not show an inverted U shape. The relationship between the two shows an ascending curve with no obvious inflection point. This is not the same as in Xie et al. [8] and others who studied the relationship between PM_2.5_ and economic growth, which verifies that the effect of controlling Nitrogen Dioxide after 2013 is not ideal. The reason is that, even though some developed cities have entered a stage where low energy consumption industries account for a relatively high proportion, the purchasing power of urban residents for automobiles and the penetration rate of natural gas use and residential use will increase with economic growth, and China is only in the process of controlling automobile exhaust. The technical level of Nitrogen Dioxide emissions has not reached a very mature stage. In addition, it is very difficult to introduce more advanced natural gas equipment into every household in each city with a low emission rate. At the same time, the ability to treat Nitrogen Dioxide in industrial production is also lower than that of carbon dioxide, PM_2.5_ and other waste gas. The estimated results of the spatial autocorrelation coefficient and the control variable coefficient are shown in Table 4.

The estimated value of *ρ* is 0.2580, which is significant at the 1% level. This result is consistent with the test result of the Moran analysis. It shows that the concentration of Nitrogen Dioxide has significant spatial autocorrelation characteristics and a significant spatial spillover effect. A 1% change in the concentration of Nitrogen Dioxide in neighboring cities will result in a change of 0.2580% in the concentration of Nitrogen Dioxide in the local city. The estimated value of *ρ* has the same characteristics as the value of the Moran index, and both are higher than zero, which once again confirms that the distribution of Nitrogen Dioxide concentration in different urban areas has positive spatial aggregation characteristics. In China, such as Jiangsu and Hebei provinces with heavy industries and large populations, the neighboring urban areas within the provinces usually have similar air pollution levels, which provides a practical basis for the spatial accumulation of Nitrogen Dioxide.

First, the estimated coefficient of PCG is 0.4249, which is significant at the 1% level, indicating that economic growth has a positive effect on changes in Nitrogen Dioxide concentration. For every unit increase in PCG, the concentration of Nitrogen Dioxide will increase by 0.4249. On the one hand, economic growth requires increased investment, which in turn increases the use of resources; on the other hand, more output also brings about an increase in the emission of Nitrogen Dioxide, which is air pollution. The scale effect worsens the pollution of Nitrogen Dioxide, while the technical and structural effects improve this problem. In the current stage of China’s economic development, the scale effect exceeds the technical and structural effects, resulting in the inability to alleviate Nitrogen Dioxide pollution. This is also consistent with the regression results in Figure 4. At present, the relationship between China’s per capita GDP and Nitrogen Dioxide pollution shows a linear increase, and the inflection point does not appear.

Secondly, the estimated coefficient of green coverage is −0.2151, which is significant at the 1% level, indicating that green coverage has a negative impact on the concentration of Nitrogen Dioxide. Green vegetation has played a part in the process of reducing Nitrogen Dioxide pollution. Oksanen and Kontunen-Soppel [24] pointed out that due to the different metabolic reactions of different plants and the interaction with other co-existing air pollutants, although the physiological response of nitrogen oxides is complex, some plants can absorb Nitrogen Dioxide through stomata or deposits on the surface of leaves. However, we must be vigilant in that, although urban green coverage now has an impact on the reduction of Nitrogen Dioxide concentration, with the advancement of urbanization, the green coverage rate of most cities may decline, which means that green vegetation can inhibit the effect and urban Nitrogen Dioxide concentration will be reduced. It can also be calculated from the data that, taking 2018 as an example, the urban green coverage rate has increased by an average of 0.37% compared with 2017, which is barely maintained at a positive value.

Third, the estimated coefficient of industrialization is 0.1389, which is significant at the 1% level, indicating that industrialization has a positive correlation with the concentration of Nitrogen Dioxide. The proportion of secondary industry represents the level of industrialization of a city. The secondary industry in Chinese cities is clearly related to the production of many air pollutants. Xie et al. [8], Wu et al. [9] and others have previously confirmed that there is a significant positive correlation between PM_2.5_ emissions and the level of urban industrialization. In industrial production, a large amount of Nitrogen Dioxide is emitted during the production process of many industries. Mele and Magazzino [25] pointed out that, in the iron and steel manufacturing process, sintering, pelletizing, and hot-blast stove combustion will generate a large amount of Nitrogen Dioxide. A large amount of Nitrogen Dioxide is formed by the combustion of Nitrogen Dioxide in the cement-making process and the decomposition of raw materials during high-temperature processing.

Fourth, the estimated coefficient of transportation development is 0.0636, which is significant at the 1% level. This data shows that traffic development has a positive impact on the concentration of Nitrogen Dioxide. Economic growth will make people wealthy, and China is currently in the developing stage, and more and more people have the ability to buy cars, which has led to a very high increase in the number of urban civilian cars in China. According to data calculations, in 2018 annual car growth reached 11.76%. More and more cars have caused more Nitrogen Dioxide emissions, accelerating urban Nitrogen Dioxide pollution.

Fifth, the estimated coefficient of investment in science and technology is −0.0003. Although this investment has a negative impact on the concentration of Nitrogen Dioxide, the result is not significant, because it includes too many aspects, and investment in science and technology for the treatment of Nitrogen Dioxide only accounts for a small part. Investment in science and technology also includes investment in promoting production and investment in environmental governance. The ratio of the two and the degree of mutual influence are different in different cities. This has caused a certain interference in the data analysis. Therefore, investment in science and technology does have an impact on the concentration of Nitrogen Dioxide, though the impact is very vague and not significant.

Sixth, the estimated coefficient of natural gas consumption is 0.0581, which is significant at the 1% level. This data shows that the use of natural gas has a positive correlation with the concentration of Nitrogen Dioxide. Natural gas itself does not produce Nitrogen Dioxide, but it will be produced during high-temperature combustion. The use of natural gas does not only come from the factor of residential natural gas consumption. It relates to a wide range of applications, including natural gas power generation, natural gas chemical industry, and compressed natural gas vehicles. These applications are the products of the energy substitution policy, which reduces the consumption of coal and gasoline, thereby ensuring the reduction of sulfide and carbon dioxide emissions, but loses sight of other objectives, resulting in a further increase in Nitrogen Dioxide emissions.

Seventh, the estimated coefficient of residential natural gas consumption is 0.0405, which is significant at the 5% level. This data has the same performance as natural gas consumption, and both have a positive effect on the concentration of Nitrogen Dioxide. Residents’ natural gas consumption reflects to a large extent the contribution of the permanent population of each city to the concentration of Nitrogen Dioxide. Residents cannot do without the use of natural gas in their daily life, for cooking, heating, and bathing. Especially in winter, people’s heating demand is very large, and it can also be seen from the data that the concentration of Nitrogen Dioxide in each city in winter has increased significantly.

### 5.2. Robustness Analysis

#### 5.2.1. Estimated Results of SEM Model

Although this research is based on the estimation results of the spatial lag model, the LM and LR test results show that there are both spatial error effects and spatial lag effects. When this happens, a spatial error model must be introduced on the basis of Equation (3), and the spatial autocorrelation coefficient of Equation (6) and the coefficient of each control variable must be compared to ensure the rigor of the spatial autoregressive research method. The expression of the spatial error model is

(7) $$lnNO_{2i}=a_{1}lnPCG+a_{2}lnPCG^{2}+B_{1}lnSI_{i}+B_{2}lnIT_{i}+B_{3}lnGC_{i}+B_{4}lnVN_{i}+B_{5}lnNG_{i}+B_{6}lnRNG_{i}+u$$

(8) $$u=ƛW_{ij}u+e$$

ƛ is the spatial autocorrelation coefficient, also known as the spatial autocorrelation error term. *u* is the error term.

It can be seen from Table 5 that per capita GDP, industrialization, transportation development, natural gas consumption, and residential natural gas consumption all have a positive effect on the increase of Nitrogen Dioxide concentration. Green coverage has a negative impact on the concentration of Nitrogen Dioxide. The impact of technology investment is not significant. This is consistent with the estimation results of the spatial lag model.

#### 5.2.2. Robustness Analysis of Dynamic QML

A robustness test can solve the problems of unreliable parameter standard errors caused by heteroscedasticity, sequence autocorrelation and endogenous problems, so it is very necessary to test whether the results of empirical analysis maintain appropriate robustness with changes in parameter setting,

Table 6 shows the QML estimation results of the dynamic system. In the estimation specification, the concentration of Nitrogen Dioxide, lagging one year behind, is used as an additional explanatory variable to reflect the influence of man-made changes in the concentration of nitrogen dioxide and the dynamic characteristics of Nitrogen Dioxide concentration accumulation in the previous year. The results compared with the conclusion of the spatial lag model show that although the coefficients of the variables are slightly different, per capita GDP, industrialization, transportation development, natural gas consumption, and residential natural gas consumption still have a positive effect on the increase of Nitrogen Dioxide concentration. Green coverage has a negative impact on the concentration of Nitrogen Dioxide. The impact of technology investment is not significant. The *p*-value of the AR2 test of the model is much larger than 0.1, indicating that there is no second-order serial correlation in the first differential perturbation. Therefore, the result is valid.

### 5.3. Long-Term Effects Based on Dynamic QML Test

It can be seen from Table 6 that in terms of long-term effects, the impact of green coverage on the concentration of Nitrogen Dioxide will continue to maintain a negative correlation, and the coefficient will become smaller. This is satisfactory in the long run, indicating that greening has a positive effect on the concentration of Nitrogen Dioxide. The reduction in the concentration of Nitrogen Dioxide will become more and more obvious over time, and it takes a longer time for green plants to perform their purification function. This conclusion is supported by Wu et al. [9]. However, in terms of the long-term effects of industrialization, transportation development, and natural gas consumption, the respective coefficients have almost doubled compared with the previous analysis results. For example, in the future, the proportion of urban secondary industries will increase by 1%. The nitrogen concentration will rise by 0.26%. This shows that if urban development does not control the changes in the proportion of the secondary industry, does not control the number of urban cars, and relies too much on natural gas to replace coal, oil and other energy sources, it will be difficult to effectively control Nitrogen Dioxide pollution in the future. Residents’ natural gas consumption coefficient has also risen slightly, and technological input still has no significant impact on the control of Nitrogen Dioxide concentration in the long-term effect.

## 6. Conclusions

Based on the data of 333 cities in China from 2016 to 2018, this study studied the impact of economic growth, industrialization, technology investment, green coverage, transportation development, natural gas consumption, and residential natural gas consumption on the concentration of Nitrogen Dioxide. Because the traditional regression function form is not rigorous enough, the commonly used parameter models will lead to estimation bias and inconsistency. In order to overcome this problem, this paper uses the spatial autoregressive model as an econometric method to study the spatial spillover effect of Nitrogen Dioxide pollution and the nonlinear relationship between economic growth and Nitrogen Dioxide concentration. The results show that the concentration of Nitrogen Dioxide presents positive spatial accumulation characteristics. There is a slowly increasing nonlinear relationship between economic growth and Nitrogen Dioxide concentration. This does not support the EKC hypothesis. There is no obvious critical point of the U-shaped curve. China has not entered the stage of sustainable development on the issue of Nitrogen Dioxide pollution. The results of the spatial lag model show that per capita GDP, industrialization, transportation development, natural gas consumption, and residential natural gas consumption all have positive effects on the increase of Nitrogen Dioxide concentration to varying degrees, while green coverage has a negative correlation with Nitrogen Dioxide concentration. The coefficient is −0.2151. The impact of technology investment is not significant.

Based on the above conclusions, the following policy recommendations are put forward.

The research results show that economic growth continues to aggravate Nitrogen Dioxide pollution, with a positive coefficient of 0.4249. It indicates that the current economic growth model may not have economic-ecological sustainable development, and the emission level will accelerate in the future as income increases. As a policy hint, (1) In terms of secondary industry, technological innovations need to be made in some aspects of the treatment of nitrogen oxide emissions in its links with industrial processing. For example, the iron and steel industry can adopt an activated carbon (with independent denitrification section) or SCR (selective catalytic reduction) process for denitration; hot blast stoves and heat treatment furnaces can adopt low-nitrogen combustion technologies such as staged combustion, delayed cycle combustion, and flameless combustion. The cement industry can popularize the installation of high-efficiency low-nitrogen burners and SNCR out-of-stock equipment and other pollution control measures. (2) In terms of transportation development, there is a need to optimize transportation development methods, improve transportation efficiency, encourage the introduction of new energy vehicles in the market, and guide residents towards green travel, so as to achieve emission reductions in the transportation system. (3) In terms of natural gas consumption, the energy substitution policy of compressed natural gas vehicles should be reduced as much as possible, the promotion of electric cars should be encouraged, and natural gas equipment in residents’ homes should be upgraded to reduce nitrogen emissions. (4) The green coverage area should be higher than the newly planned land area for development in the city in terms of growth rate to ensure the sustainability of the green environment. (5) In terms of scientific and technological investment, it is necessary to clarify and increase scientific research funding for Nitrogen Dioxide treatment in order to embark on the goal of long-term sustainable development.

Nitrogen Dioxide pollution has a strong positive spatial spillover effect. As a policy hint, multi-regional joint measures need to be taken to prevent Nitrogen Dioxide from migrating in a certain region and interacting with others. Specifically, for the high-energy mining Fenwei Plain, the fast-developing Yangtze River Basin, and the Beijing-Tianjin wing area, the regional control mechanism should be optimized, and special projects should be established to treat Nitrogen Dioxide. To establish an overall prevention and control system to promote the treatment of Nitrogen Dioxide in cities in the region, it is suggested that the government should guide all cities to work together in the future to help reduce pollution in the region.

Of course, there are still many problems worthy of in-depth discussion in this study. For example, the geographical centroid distance of some adjacent cities investigated in this paper is too far, and the 01 matrix is not as good as the inverse distance matrix in this regard. If it is replaced by the inverse distance matrix, will the result be more accurate? Chinese residents use a lot of natural gas for heating in winter, and the combustion of natural gas will produces a lot of nitrogen oxide. If the winter heating months in the sample data are extracted separately for analysis, will the impact of natural gas on Nitrogen Dioxide pollution change, or by how much will it increase?

## Figures and Tables

**Table 1:** Lagrange multiplier test results.

Test	Statistic	Df	*p*-Value
Spatial error:			
Moran’s I	2.452	1	0.014
Lagrange Multiplier	115.725	1	0.000
Robust Lagrange multiplier	102.723	1	0.000
Spatial lag:			
Lagrange Multiplier	24.478	1	0.000
Robust Lagrange multiplier	11.477	1	0.001

**Table 2:** Likelihood-ratio test results.

lrtest Sdm Sar	
Likelihood-ratio test	LR chi2(8) = 12.78
(Assumption: sar nested in sdm)	Prob > chi2 = 0.1198
lrtest sdm sem	
Likelihood-ratio test	LR chi2(8) = 12.24
(Assumption: sem nested in sdm)	Prob > chi2 = 0.1408

**Table 3:** Descriptive statistics.

Variable	Definition	Units of Measurement	Mean	Std. Dev.	Min	Max
NO_2_	NO_2_ concentrations	µg/m^3^	36.33	12.68	6.88	71.80
PCG	Per capita GDP	yuan	60,363.98	34,059.83	10,707	215,488
VN	Traffic development	Number of vehicles	720,129.5	774,805.8	22,361	5,631,000
SI	Industrialization	%	43.29	10.25	9	72.9
GC	Green coverage	%	39.51	6.07	3.07	67
NG	Nature gas consumption	10,000 m^3^	37,456.16	122,005.7	0	1,915,978
IT	Technology investment	%	0.43	0.54	0.01	6.31
RNG	Nature gas consumption of residents	10,000 m^3^	8075.53	19,032.59	0	203,411

**Table 4:** The estimated result of the spatial lag model.

Variable	*ln PCG*	*ln GC*	*ln SI*	*ln VN*	*ln IT*	*ln NG*	*ln RNG*	* ρ *
Coef.	0.4249 ***	−0.2151 ***	0.1389 ***	0.0636 ***	−0.0003	0.0581 ***	0.0405 **	0.2580 ***
Std.dev.	0.3799	0.0533	0.0374	0.0127	0.0109	0.0123	0.0131	0.0317
Z	2.17	−4.03	3.72	5.00	−0.03	4.74	3.09	8.15

Note: *, **, *** indicate statistical significance at the 10%, 5%, and 1% level, respectively.

**Table 5:** Estimated results of the spatial error model.

Variable	*ln PCG*	*ln GC*	*ln SI*	*ln VN*	*ln IT*	*ln NG*	*ln RNG*	*ƛ*
Coef.	0.3651 ***	−0.1402 **	0.0927 *	0.0765 ***	0.0598	0.0692 ***	0.0394 **	0.4217 ***
Std.dev.	0.2943	0.0514	0.3838	0.0138	0.0116	0.0121	0.0129	0.0368
Z	1.44	−2.73	2.42	5.56	0.51	5.73	3.03	11.46

Note: *, **, *** indicate statistical significance at the 10%, 5%, and 1% levels, respectively.

**Table 6:** Robustness analysis results of dynamic QML.

	*L.WlnNO* *_2_*	*lnPCG*	*lnPCG^2^*	*lnGC*	*lnSI*	*lnVN*	*lnIT*	*lnNG*	*lnRNG*	*AR2*
Main	0.3894 ***	0.2550 *	−0.0138	−0.2969 ***	0.1580 ***	0.0742 ***	0.0012	0.0558 ***	0.0343 *	0.501
LR.		0.3808 *	−0.0207	−0.4955 ***	0.2608 ***	0.1241 ***	0.0019	0.0916 ***	0.0582 *	

Note: *, **, *** indicate statistical significance at the 10%, 5%, and 1% levels, respectively.

## Data Availability

All data and material generated or used during the study appear in the submitted article.

## Abbreviations

*NO_*2*_*	Nitrogen dioxide concentrations
*PCG*	Per capita GDP
*VN*	Traffic development
*SI*	Industrialization
*GC*	Green coverage
*NG*	Nature gas consumption
*IT*	Technology investment
*RNG*	Nature gas consumption of residents
*EKC*	Environmental Kuznets Curve
*QML*	Quasi-maximum likelihood
*CNEMC*	China Environmental Monitoring Center
*CCSY*	China City Statistical Yearbook
*SCR*	Selective Catalytic Reduction
